# Sodium Content of Processed Foods Available in the Mexican Market

**DOI:** 10.3390/nu10122008

**Published:** 2018-12-19

**Authors:** Claudia Nieto, Lizbeth Tolentino-Mayo, Catalina Medina, Eric Monterrubio-Flores, Edgar Denova-Gutiérrez, Simón Barquera

**Affiliations:** Centro de Investigación en Nutrición y Salud, Instituto Nacional de Salud Pública, Cuernavaca 62100, Mexico; claudia.nieto@insp.mx (C.N.); mltolentino@insp.mx (L.T.-M.); catalina.medina@insp.mx (C.M.); eric@insp.mx (E.M.-F.); edgar.denova@insp.mx (E.D.-G.)

**Keywords:** sodium, sodium targets, food industry, diet

## Abstract

Background: Sodium intake is related to several adverse health outcomes, such as hypertension and cardiovascular diseases. Processed foods are major contributors to the population’s sodium intake. The aim of the present study was to determine sodium levels in Mexican packaged foods, as well as to evaluate the proportion of foods that comply with sodium benchmark targets set by the United Kingdom Food Standards Agency (UK FSA) and those set by the Mexican Commission for the Protection of Health Risks (COFEPRIS). We also evaluated the proportion of foods that exceeded the Pan American Health Organization (PAHO) targets. Methods: This was a cross-sectional study that comprised data collected from the package of 2248 processed foods from selected supermarkets in Mexico. Results: Many processed food categories contained an excessive amount of sodium. Processed meats, ham, bacon and sausages, had the highest concentrations. The proportion of foods classified as compliant in our sample was lower for international targets (FSA UK and PAHO) compared to the Mexican COFEPRIS criteria. Conclusions: These data provided a critical baseline assessment for monitoring sodium levels in Mexican processed foods.

## 1. Introduction

Sodium intake is related to several adverse health outcomes, such as hypertension, cardiovascular diseases, and death [1,2,3,4]. In 2010, approximately 1.65 million cardiovascular deaths in the world were attributed to a salt intake above the limit of 5 g a day [3] and in some areas in North/South America, this was the 9th to 15th leading cause of premature death. In Mexico, the prevalence of hypertension in adults reached 31.5% [5]. Furthermore, cardiovascular diseases were the first cause of death in the country [6]. The World Health Organization (WHO) recommends that the intake of salt should be less than 5 g per day [7]. In 2013, the Global Action Plan for the Prevention and Control of Non-Communicable Diseases set a target to reduce the population intake of sodium by 30% [8], since it is considered one of the most cost-effective interventions to improve population health [9]. Due to sodium’s effect on the population’s health, several countries have introduced strategies to reduce it, including health promotion campaigns, taxes, food labelling, consumer education, and public health interventions [10,11]. In Mexico, some strategies like removing saltshakers from tables of restaurants and reducing sodium in bread have been implemented [12,13].

Processed foods are major contributors to the population’s dietary salt intake [14,15,16,17]; therefore, lowering sodium in packaged foods can be an important intervention to reduce it. In the Mexican population, the main dietary sources of sodium are breads, meats, pizzas, sandwiches, cheese, and some packaged foods such as soups, rice, and snacks [18]. A recent study found that ready to eat breakfast cereals were high in sodium content [19]. Since 36% of the total energy intake of the Mexican diet comes from processed and ultra-processed foods [20], an assessment of current sodium content is key to monitoring processed foods and encouraging reformulation. Some institutions have been working to establish targets in order to monitor and evaluate the content of sodium. Those institutions are: The Food Standard Agency (FSA) in United Kingdom (UK), the Federal Commission for Protection against Health Risks (COFEPRIS, by its acronym in Spanish) in Mexico, and the Pan American Health Organization (PAHO) in the Pan-American region.

In this context, the UK FSA established targets for 2017 aiming for further reduction of sodium content [21]. They also recognized the progress made by the UK food industry in 2013; nevertheless, they acknowledged the potential to further reduce the salt content in processed foods with the new targets [22]. In Mexico, as a part of a policy package to fight obesity and chronic diseases, the Mexican government, specifically COFEPRIS, implemented a voluntary strategy for packaged foods. This voluntary legislation consisted of obtaining the nutritional stamp endorsed by the Ministry of Health if food manufacturers accomplish nutrients criteria. Such stamp aimed to indicate if a product is healthy for regular consumption. This legislation, approved by the Ministry of Health in 2014, established cut-off points regarding the maximum levels of energy, sodium, saturated fat, and sugar allowed in commonly consumed foods [23]. Finally, in las Americas the PAHO brought together a consortium of governments, civil society, and food companies (the Salt Smart Consortium) to set maximum targets (upper limits) for sodium levels for 11 food categories to be achieved by December of 2016. The technical advisory group (TAG) used their experiences and lessons learned to provide guidance on establishing national initiatives that encourage food companies to reformulate their products [24]. The food categories considered were: Bread, soups, mayonnaise, biscuits and cookies, cake, meats, breakfast cereals, cheese, processed cheese products, and cheese spreads, butter/dairy spreads and margarine, snacks, pasta, and condiments.

To date, Mexico does not have a monitoring system to evaluate the sodium content of processed foods. Furthermore, Mexico does not have an assessment that shows compliance with international, regional, and local targets. Thus, the main objectives of the study were to determine sodium levels in Mexican packaged foods and to evaluate the proportion of foods that comply with sodium benchmark targets set by the UK FSA and COFEPRIS. We also evaluated the proportion of foods that exceeded the PAHO targets.

## 2. Materials and Methods

### 2.1. Study Design

This cross-sectional study comprised data collected from July to December of 2015. Data were collected from selected supermarkets in the country.

A sampling strategy was used to identify the largest and most densely populated cities in the country (Mexico City, Guadalajara, Queretaro, Monterrey, Hermosillo, and Cuernavaca) [25]. Our sample frame was based on a list of all supermarkets in each city, and those supermarkets were mapped using a geo-reference system to determine the Basic Geostatistical Areas (AGEB by its acronym in Spanish) where they were located. AGEB are a proxy-estimation of socio-demographic characteristics of the area in each city, which delimitates urban areas with 25,000 inhabitants or more, and are used to identify specific conditions such as living, commercial, and industrial usage. The supermarkets in each AGEB were randomly and proportionally selected to the distribution of three levels of marginalization defined by the National Institute of Statistics and Geography (low, middle and high) [26]. The visited stores were hypermarkets, supermarkets, membership food stores, and convenience stores. Those stores were: 7 Eleven, Walmart, Bodega Aurrera, Bodega Express, Chedraui, City market, Comercial Mexicana, Cotsco, Extra, Fresko, Ley, Mega Comercial, Mini-super, Oxxo, Sams, Santa Fe, Smart, Soriana, Sumesa, Superama, Vimark, Waldos, and grocery stores that are not part of a franchise. Those together represent approximately 70% of the Mexican market share [27]. We did not have any store losses since the permits were given by the legal department of each main food retail chain prior to the data collection. All available food products at the time in the stores’ aisles were included. This sampling allowed an extensive coverage of available food products in Mexico.

Photographs of the labelling and package of processed foods were taken from main food retail chains in the country. One fieldwork coordinator, four trained fieldworkers with broad experience in survey data collection, and five nutrition students were trained by researchers of the Mexican National Institute of Public Health following the International Network for Food and obesity/non-communicable diseases, Research, Monitoring and Action support (INFORMAS) protocol of food labelling [28]. The personnel who collected the data followed a standardized operation procedure according to Kanter et al. [29].

Nutrition content information from photographs were captured into an excel spreadsheet. The fieldworker coordinator revised the completeness and accuracy of the data. We randomly selected a subsample of 300 products to check the captured data against the photographs taken. The database included the following information: Product name, brand, price, claims, serving size, nutrition content, and location of supermarket. In the case of exact duplicates, the most recently entered product was used. Information from (*n* = 2248) different food products was analyzed. Sodium content was recorded in mg per portion and then converted into mg/100 g. Food categories and subcategories were defined based on the UK FSA and on the PAHO criteria. The UK FSA targets for sodium were lower compared to the COFEPRIS targets. Some examples are: Ham (650 mg sodium vs. 800 mg sodium), breakfast cereals (235 mg sodium vs. 500 mg sodium), and biscuits (220 mg sodium vs. 450 mg sodium). There are two types of averages used within the UK FSA targets. The first is a processing average (p) that is used to account for ranges of salt levels that occur in a single product (e.g., bacon and mozzarella cheese). The second is a range average (r) which is used to account for a range of different flavors (e.g., standard potato crisps) covered by a single target. All range averages were calculated on a sales weighted basis. Reduced mayonnaise refers to those mayonnaises that are reduced fat or reduced calories; nevertheless, COFEPRIS does not have a target for reduced mayonnaise [21]. Similarly, PAHO targets were lower compared to COFEPRIS Mexican targets. The PAHO has supported sodium reduction policies in the Americas and proposed inaugural regional targets for several food categories in 2014 [24]. These targets were based on national targets for sodium reduction in the region, either voluntary or regulatory. A more stringent target was also proposed, based on the lowest targets in the region, in order to support countries with their national sodium reduction policies and to improve their current policies [30].

### 2.2. Statistical Analysis

The database was imported to STATA format to be cleaned. First, we identified outliers of the sodium content by each food category or subcategory. When an extreme value was found, we checked against photographs of processed products to see if the value was correct. Additionally, we randomly checked the sodium content against the photographs of the products to ensure accuracy.

First, normal distribution of the variables was calculated. Mean and standard deviations of sodium content (mg/100 g) of food categories and subcategories were calculated. Percentiles were also calculated since most of the data were skewed. We calculated the proportion of compliant food products by the FSA benchmarks and COFEPRIS cut-off points when available. Differences in the proportion of compliant food categories and subcategories between the UK FSA targets and COFEPRIS criteria were explored using tests of proportions. For all the analyses, significance was established when *p* < 0.05. All analyses were performed using STATA version 14 (StataCorp, College Station, TX, USA).

### 2.3. Ethical Approval

This study was evaluated and approved by the Research, Ethics and Biosafety Committees of the National Institute of Public Health of Mexico (ethical approval number: 1275). Before conducting the study, the research team asked for permission from the supermarket manager to access the stores and take photos of processed foods available.

## 3. Results

This analysis included 2248 food items from 12 food groups. Table 1 shows the mean sodium content in mg per 100 g. The food groups with the highest sodium content were: Ham (1255.1 mg/100 g), bacon (1027.4 mg/100 g), sausages (883.9 mg/100 g), reduced mayonnaise (868.9 mg/100 g), processed cheese (862.7 mg/100 g), and mayonnaise (751.7 mg/100 g). There was high variability in sodium levels across several product categories including: Soups (220.0–5165.7 mg/100 g), pasta (4.2–3480.0 mg/100 g), and biscuits (4.0–2778.8 mg/100 g). In contrast, there was less variability in the sodium content of standard potato crisps (400.0–560.0 mg/100 g) and mozzarella cheese (303.64–674.0 mg/100 g). Butter and cake had the lowest sodium content with 129.7 mg/100 g and 263.1 mg/100 g respectively.

**Figure 1:**
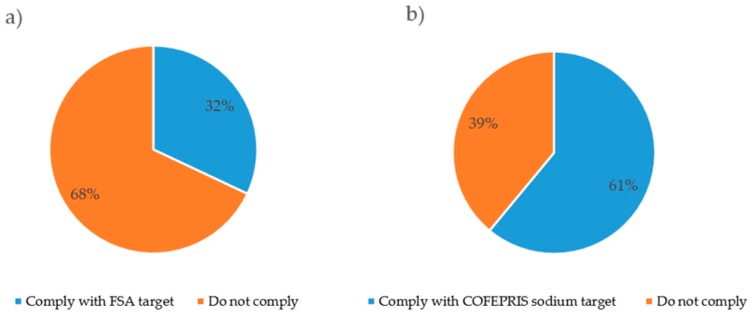
(**a**) Proportion of packaged foods meeting and exceeding the Food Standard Agency (FSA) sodium benchmark targets. (**b**) Proportion of packaged foods meeting and exceeding the Federal Commission for Protection against Health Risks (COFEPRIS by its acronym in Spanish) sodium targets (*n* = 2248).

Processed foods in the Mexican market were also classified as compliant and non-compliant according to two profiling systems: The UK FSA targets and the COFEPRIS criteria. Overall, 61% complied with COFEPRIS target, while only 32% of foods complied with the FSA target (Figure 1). In other words, twice the amount of food products complied with the COFEPRIS target than with UK FSA criteria.

Table 2 shows the proportion of packaged foods that comply with sodium targets from the UK FSA and COFEPRIS. The highest proportion of foods meeting the UK FSA targets were butter (93%), salt and vinegar crisps (71%), and bacon (62%), whereas mayonnaise (0%), reduced mayonnaise (0%), and soups (2%) had the lowest compliance. On the other hand, the highest proportion of foods meeting the COFEPRIS criteria were mozzarella cheese (100%), fresh cheese (94%), and butter (93%). The lowest compliance levels were for sausages (22%), soups (24%) and ham (28%).

### 3.1. Comparison UK FSA vs. COFEPRIS

From the 43 types of ham collected, 14% complied with the UK FSA target (650 mg of sodium/100 g), while 28% complied with COFEPRIS (800 mg of sodium/100 g). Sausages faced a similar situation; 7% complied with the FSA target and 22% complied with COFEPRIS. No statistically significant differences were found for those two subgroups (*p* > 0.05). Among the different kinds of bacons assessed, only 38% of different bacons are above the UK FSA target whereas COFEPRIS does not have a cut-off point. Bread had 14% of products complying with UK FSA targets compared to 61% complying with COFEPRIS criteria (*p* < 0.001). For breakfast cereals, 37% complied with UK FSA, while 78% complied with COFEPRIS (*p* < 0.001). For mayonnaise and for reduced mayonnaise none of the products complied with the UK FSA target, while the proportion of mayonnaise that complied with the COFEPRIS criteria was 59%. The only food subgroup that had the same proportion of compliance for both targets was standard potato crisps (60%). Even though cakes had one of the lowest mean sodium contents, only 23% complied with UK FSA target (170 mg of sodium/100 g) and 89% complied with COFEPRIS sodium criteria (450 mg of sodium/100 g) (*p* < 0.001) (Table 2).

### 3.2. PAHO Sodium Reduction Targets

Finally, Table 3 shows the food categories and subcategories that exceed the regional and lower targets set by the PAHO. Soups were the category with the highest proportion above the regional target (73%), while butter complied the most with 100% of the regional target established by PAHO. Meats were the category with the highest proportion above the lower target (91%). Butter only had 8% above the lower target. Snacks and breads also had great proportions above the PAHO regional target, 35% and 29% respectively. Soups and snacks had great proportions above the lower target, (88% and 83%, respectively). The food categories that complied the most with the regional targets were: Butter (100%), meats (98%), and breakfast cereals (96%). However, lower targets were harder to meet, being butter (92%), breakfast cereals (78%), and pasta (77%) who came closest to meeting the targets.

## 4. Discussion

Many processed food categories contained an excessive amount of sodium. Processed meats (ham, bacon, and sausages) had the highest concentrations. These data are consistent with the SALMEX study that found processed meat was the main contributor to daily sodium intake, representing 8% of total sodium intake per capita measured by three-day food records [31]. In the sample studied we found that the proportion of foods classified as compliant was lower for international targets (UK FSA and PAHO) compared to the Mexican standards established by COFEPRIS. Finally, to our knowledge, this is the first paper that evaluates and monitors the sodium content of processed foods in Mexico. In general, the maximum sodium content in processed foods established by international (UK FSA) and regional (PAHO) agencies is lower than the levels suggested by COFEPRIS in Mexico. Nevertheless, sodium content in processed foods is high and we should aim to meet the WHO recommendation.

This evidence might encourage the utilization of regional and international targets to monitor and evaluate the progress made by the food industry. As part of the policy package to stop the epidemic of diet-related diseases, like hypertension and cardiovascular diseases, the Mexican food stamp (COFEPRIS criteria) should identify products high in sodium content. Nevertheless, we found statistically significant differences in the proportions of foods complying with FSA targets and COFEPRIS criteria. This might be partially explained by the close participation of the food industry in the design of nutrient profiling systems. In the past, the food industry has been invited to participate in committees that make food policy decisions. A case study recently documented such interference in the profiling system of the Mexican front of package labelling [32].

Since the compliance is easy to meet, the current strategy does not promote food reformulation. The Mexican government could reduce the cut-off points of the nutritional stamp to promote food reformulation by food manufacturers. In this sense, the definition of new maximum levels of sodium in processed food could contribute to the reduction among the Mexican population. In the Mexican adult population, it is known that processed and ultra-processed foods contribute 36% to total energy [33]; nevertheless, an estimate of how much of the sodium intake these products contribute to the average sodium intake is lacking. In Australia, evidence shows that ultra-processed food provides 40% of sodium in preschool children [34]. In United States, quick service restaurants that mostly serve processed and ultra-processed foods provide 8% of total sodium in adults diets [35].

Despite the existence of Mexican voluntary targets, experience has proved that without government surveillance and regulation there is not a sufficient incentive for the food manufacturers to reformulate products [36]. Ultimately, mandatory targets for processed foods will be needed to substantially reduce sodium dietary intake across the Mexican population. A gradual transition to stringent profiles such as the PAHO benchmarks is recommended. Setting targets is feasible; a number of countries in the Pan-American region like Argentina, Brazil and Canada have implemented timelines for food reformulation [24]. Besides, existing food technology can help to maintain taste when reducing the sodium content [37]. Furthermore, after the reformulation, it is important to monitor adherence to targets; this monitoring system should be transparent and regularly verified [38]. Public education and social marketing are also needed to motivate the population to choose a healthier diet with a lower sodium content [39]. Afterwards, the demand for low and sodium free products is expected to rise. For example, hypertensive older adults who are conscious about the health consequences of salt had higher willingness to consume low-sodium options [40]. Furthermore, a study documented that the majority of consumers agree that it is a good idea for governments to restrict food manufacturers from putting excess salt in foods [41]. Another strategy with a population approach to reduce sodium intake is the front of package labelling. Uruguay and Chile, for example, have a warning labelling system that is easily understood by the population, which helps consumers make healthier food choices [42]. Besides, Chile’s criteria are stringent because it was based on evidence. The implementation of their front of package labelling system had a plan to implement progressive thresholds to move closer to PAHO criteria [11]. The local government of Mexico City has taken some steps toward reducing sodium intake. There is a local strategy that aims to reduce it: The campaign “Less salt, more health,” which removed saltshakers from tables of restaurants. In a recent evaluation, 5179 restaurants followed the campaign [12]. One of the limitations of the strategy is that the daily consumption of sodium cannot be tracked; therefore, it is hard to prove that removing saltshakers from tables is effective. Future assessments of this strategy are highly desirable. Another effort is the national agreement to reduce 10% of the sodium content of bread [13]. This voluntary agreement was implemented during 2012; however, an evaluation of this public health measure has not been conducted.

This study used data taken from the package and labelling of processed foods and does not assess individual sodium intake. In Mexico, the surveillance of food labelling is undertaken by the Ministry of Health along with COFEPRIS. The accuracy of the nutrition information displayed in packages is regularly checked with bromatological studies that assess the food composition in order to verify that the information is consistent with the actual content of foods [43]. Furthermore, open-access food composition data provided by the food industry would simplify efforts to monitor and assess the content of food products and their nutrients of concern. This study was cross-sectional; therefore, it does not evaluate the progress in reformulation. In future studies, data from different years will be needed to assess the reformulation of the nutrition content. Research is needed to assess the national and local initiatives, evaluate the population’s sodium dietary intake, and identify the contribution of processed and ultra-processed foods to the diet.

### Global Implications

The estimated mean level of global sodium consumption is 3.95 g per day. Globally, 1.65 million annual deaths from cardiovascular causes were attributed to sodium intake above the reference level [3]. Since the contribution of sodium comes from processed foods [44,45], setting and aligning targets is a global initiative that could decrease the burden of non-communicable diseases [10,11,46]. A total of 75 countries had adopted a national salt reduction strategy by 2015. Nevertheless, more efforts are needed to support low- and middle-income countries to comply with international recommendations [46].

## 5. Conclusions

These data provide a critical baseline assessment for monitoring sodium levels in Mexican processed foods. This assessment will allow for further monitoring of sodium levels and food reformulation. The majority of food groups were found to be high in sodium. Most of them are above the COFEPRIS criteria which are less stringent than the international or regional targets. Processed foods are widely consumed by the Mexican population. Given the health priorities in Mexico, our findings support that strong regulations are needed to reformulate processed foods available in the Mexican market. This measure could have the potential to decrease health risks due to high sodium intake.

## Figures and Tables

**Table 1:** Sodium content of processed food groups and subgroups available in the Mexican market (mg/100 g) (*N* = 2248).

Food Group	Subgroup	*n*	Min	Max	Mean	SD	p25	p50	p75
Meat Products	Bacon *	21	90	2133	1027	585	600	1000	1318
	Ham	43	500	2900	1255	738	745	995	1580
	Sausages	82	70	1500	884	204	807	897	982
Bread		215	133	1500	552	215	390	447	616
Breakfast Cereals	Breakfast cereals	404	0	1062	298	223	67	323	480
Cheese	Processed cheese	60	210	2667	863	421	600	780	1149
	Fresh cheese *	35	14	970	498	209	363	568	615
	Mozzarella *	17	304	674	510	147	360	570	643
Butter	Butter	40	0	740	130	231	0.7	9.02	198
Fat Spreads	Margarine	22	400	920	586	182	440	530	735
	Mayonnaise	29	536	1250	752	218	570	625	932
	Reduced mayonnaise *	12	680	1200	869	139	757	883	913
Soups		84	220	5165	723	803	350	594	690
Pizzas		51	272	934	483	119	407	473	547
Crisp and Snacks	Standard potato crisps *	5	400	560	464	88	400	400	560
	Extruded and sheeted snacks	234	41	2480	839	415	578	760	1000
	Salt and Vinegar products *	7	246	1045	572	273	389	520	821
Cakes		132	0	795	263	169	200	250	340
Biscuits		594	4	2778	297	206	162	276	388
Pasta		161	4	3480	804	827	74	643	1652

* Food groups or subgroups that had a normal distribution (*p* > 0.05).

**Table 2:** Percentage of compliance of 2248 processed foods with UK FSA targets and COFEPRIS targets.

Food Group	Subgroup	*n*	UK FSA Target (mg/100 g)	COFEPRIS Target (mg/100 g)	% of Compliance with FSA Target	% of Compliance with COFEPRIS Target	*p* value
Meat Products	Bacon	21	1150	NA	62	NA	--
	Ham	43	650 (p)	800	14	28	0.5
	Sausages	82	650 (p)	800	7	22	0.4
Bread		215	360 (r)	500	14	61	0.001
Breakfast Cereals	Breakfast cereals	404	235 (r)	500	37	78	0.001
Cheese	Processed cheese	60	650 (r)	800	32	58	0.06
	Fresh cheese	35	200 (r)	800	14	94	0.001
	Mozzarella	17	540 (p)	900	47	100	0.03
Butter	Butter	40	590 (r)	500	93	93	0.5
Fat Spreads	Margarine	22	425 (r)	500	18	50	0.67
	Mayonnaise	29	500 (max)	750	0	59	--
	Reduced mayonnaise *	12	680 (max)	NA	0	NA	--
Soups		84	210 mg (r)	350	0	24	--
Pizzas		51	500 (max)	NA	57	NA	--
Crisp and Snacks	Standard potato crisps	5	525 (r)	450	60	60	0.5
	Extruded and sheeted snacks	234	680 (r)	NA	40	NA	--
	Salt and Vinegar products	7	750 (r)	NA	71	NA	--
Cakes		132	170 mg (r)	450	23	89	0.001
Biscuits		594	220 (r)	450	36	85	0.001
Pasta		161	200 (r)	500	40	64	0.01

FSA targets: There are two types of averages used within the targets table. Average (p) used to account for ranges of salt levels that occur in a single product and average (r) which is used to account for a range of different flavors. * Reduced mayonnaise: Refers to those mayonnaises that have reduced fat or reduced calories.

**Table 3:** Percentage of 1977 processed foods that exceed the regional and lower sodium reduction targets set by the Pan American Health Organization (PAHO).

Food Category	Subcategory	*n*	Regional Target	% above the Regional Target	Lower Target	% above the Lower Target
Bread		215	600	29	400	69
Soups		86	360	73	306	88
Mayonnaise		29	1050	14	670	41
Biscuits and Cookies						
	Cookies and sweet biscuits	594	485	12	265	53
Cakes		132	400	16	205	72
Meats						
	Cooked, uncooked and processed meats and sausages	82	1210	2	690	91
Breakfast Cereals		404	630	4	500	22
Butter		40	800	0	500	8
Snacks		234	900	35	530	83
Pasta						
	Shelf-stable pasta and noodles (dry, uncooked)	161	1921	12	1333	23

PAHO: Pan American Health Organization.
